# Expression profiling of 21 biomolecules in locally advanced nasopharyngeal carcinomas of Caucasian patients

**DOI:** 10.1186/1472-6890-13-1

**Published:** 2013-01-29

**Authors:** Dimitrios Krikelis, Mattheos Bobos, Georgia Karayannopoulou, Liliana Resiga, Sofia Chrysafi, Epaminontas Samantas, Dimitrios Andreopoulos, Vassilios Vassiliou, Elisabeta Ciuleanu, George Fountzilas

**Affiliations:** 1 Department of Medical Oncology “Papageorgiou” Hospital, Aristotle University of Thessaloniki School of Medicine, Ring Road of Thessaloniki, Nea Efkarpia, Thessaloniki, PC, 56403, Greece; 2 Laboratory of Molecular Oncology, Hellenic Foundation for Cancer Research, Aristotle University of Thessaloniki School of Medicine, Thessaloniki, Greece; 3 Department of Pathology, Aristotle University of Thessaloniki School of Medicine, Thessaloniki, Greece; 4 Department of Pathology, “Ion Chiricuta” Cancer Institute, Cluj, Romania; 5 Third Department of Medical Oncology, “Agii Anargiri” Cancer Hospital, Athens, Greece; 6 Department of Radiation Oncology, Bank of Cyprus Oncology Centre, Nicosia, Cyprus

**Keywords:** Nasopharyngeal carcinoma, Caucasian patients, Immunohistochemistry, Protein-expression profiling

## Abstract

**Background:**

Since scarce data exist on the pathogenesis of nasopharyngeal carcinoma in Caucasian patients, we attempted to elucidate the responsible molecular pathways in this patient population.

**Methods:**

Formalin-fixed paraffin-embedded tumor tissue samples from 107 patients, diagnosed with locally-advanced nasopharyngeal carcinoma and treated with chemotherapy or chemo-radiotherapy, were analyzed by immunohistochemistry for the expression of the following proteins: E-cadherin, P-cadherin, Fascin-1, Cyclin D1, COX-2, EGFR, VEGF-A, VEGF-C, VEGFR-2, VEGFR-3, ERCC1, p53, p63, Ki67, MAPT, phospho-p44/42MAPK, PTEN, phospho-AKT, phospho-mTOR, and phospho-GSK-3β. EBER status was assessed by *in situ* hybridization. The majority of the cases were included in tissue microarray. All stains were performed and assessed centrally by two pathologists. The median follow-up time was 76.8 (42.3 – 99.2) months.

**Results:**

Biomolecules expressed in >90% of cases were: p53, COX-2, P-cadherin, EBER, phospho-GSK-3β, and Fascin-1. WHO II+III tumors were more frequently EBER & PTEN positive and VEGF-A negative. Advanced age was significantly associated with positive phospho-GSK-3β and ERCC1 expression; male gender with positive phospho-AKT and phospho-p44/42MAPK; and worse performance status (1 or 2) with negative Ki67, ERCC1, PTEN, and phospho-mTOR expression. Earlier disease stage was closely associated with p63, MAPT, PTEN, and Cyclin D1 positivity. Univariate Cox regression analysis highlighted Cyclin D1 as a negative prognostic factor for disease-free survival (p=0.034) and EBER as a positive one for overall survival (p=0.048). In multivariate analysis, advanced age and stage, poor performance status, and positive ERCC1 emerged as predictors of worse disease-free and overall survival, as opposed to positive phospho-mTOR. Clustering analysis defined two protein-expression groups being predictive of better overall survival (p=0.043).

**Conclusions:**

Our study is the first to examine the activation and interaction of established biomolecules and signaling pathways in Caucasian NPC patients in an effort to reveal new therapeutic targets.

## Background

Nasopharyngeal carcinoma (NPC) is unique in terms of epidemiology, pathogenesis, and physical history compared to the anatomically adjacent cancers of the head and neck area. Approaching nasopharyngeal cancer as a single entity is misleading, since it is comprised of separate histology subtypes characterized by variable radio- and chemo-sensitivity, distinct prevalence patterns and different outcome [1]. Particularly, the nonkeratinizing undifferentiated subtype (WHO III) is endemic in southern China and common in Asian and African regions; Epstein-Barr virus (EBV) is implicated as the causal agent in the vast majority of these cases, characterized by a favorable prognosis. On the contrary, the keratinizing and the nonkeratinizing differentiated variants (WHO I and II, respectively) account together for 50%–75% of NPCs in the United States [2].

Evolution of personalized medicine is widely acknowledged as the only way to overcome the inherent limitations of chemotherapy [3]. As a matter of fact, research on the development of prognostic and predictive markers in head and neck squamous cell carcinomas (HNSCC) has led to the incorporation of cetuximab, an anti-EGFR monoclonal antibody, into the contemporary therapeutic management. However, these advances are not applicable to NPC arena, for which translational research has not yielded remarkable outcomes yet. As a result, NPC is still managed as a homogeneous disease and the prognosis of patients diagnosed in advanced stage remains poor [4]. Notably, the main bulk of research efforts originate from the Far East, thus questioning the potential application of any findings to NPC patients of Caucasian origin.

Building upon these prerequisites, our group performed a comprehensive, mostly immunohistochemical (IHC), expression profiling of 21 biomolecules from tumors of locally advanced NPC (LA-NPC) Caucasian patients treated with chemotherapy or chemo-radiotherapy (CRT) in the context of a Hellenic Cooperative Oncology Group (HeCOG) randomized trial [5]. Biomolecules were selected on the basis of a wide representation of signaling pathways and processes critical in driving carcinogenesis, such as angiogenesis, cell adhesion, assembly, proliferation and differentiation, cell cycle and transcription regulation, DNA repair, microtubule assembly, and inflammation mediation.

To the best of our knowledge, this is the first report of such a broad protein expression profiling in a considerable number of Caucasian NPC patients.

## Methods

### The clinical study – ethics approval

This is a translational study in LA-NPC patients performed as an extension of a randomized trial, conducted by HeCOG [5], which tested the addition of induction chemotherapy (cisplatin 75 mg/m^2^, epirubicin 75 mg/m^2^ and paclitaxel 175 mg/m^2^ every 3 weeks) (patient Group A) to the standard approach of concurrent chemo-radiotherapy with cisplatin (patient Group B). The main eligibility criteria were as follows: (i) biopsy-proven, previously untreated WHO type I, II or III NPC; (ii) age >15 years; (iii) stage II–IVB according to the American Joint Committee on Staging of Cancer classification (AJCC 7th edition, 2009); (iv) measurable or evaluable disease; (v) no other primary tumors; (vi) performance status (PS) of 0–2 according to the Eastern Cooperative Oncology Group (ECOG) scale.

Tissue paraffin blocks and peripheral blood for DNA analysis were prospectively collected from each patient registered in the study. The clinical protocol and collateral translational research studies were approved by the HeCOG Protocol Review Committee, the Institutional Review Boards in participating institutions and the Bioethics Committee of the Aristotle University of Thessaloniki School of Medicine under the general title: “Investigation of putative predictive or prognostic biomarkers in patients with locally advanced nasopharyngeal carcinoma”. The study was also registered at the Australian New Zealand registry (ACTRN 12609000730202). Prior to randomization each patient provided a study-specific written informed consent and optionally a separate informed consent for the provision of biological material for future research studies.

### Tissue samples for molecular analysis

**Figure 1:**
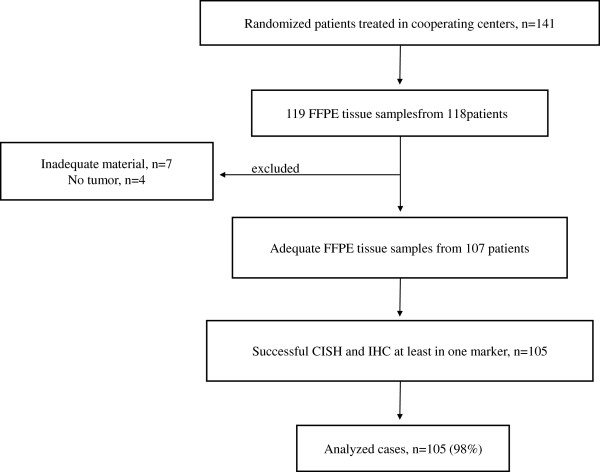
REMARK diagram of the study.

One hundred and nineteen formalin-fixed paraffin-embedded (FFPE) tumor tissue samples from 118 patients were available for analysis (only in one case, 2 samples were examined from 1 patient). Eleven blocks were not further evaluated due to inappropriate material (inadequate material in 7 cases and no tumor in 4 cases). Thus, 108 samples from 107 patients were examined for EBV-related small RNA (EBER) detection with the use of chromogenic *in situ* hybridization (CISH) [6] and for a number of key regulatory proteins by utilizing IHC [7,8]. Finally, 105 samples from 105 patients were successfully assessed with CISH and IHC (REMARK diagram, Figure 1). Representative slides (hematoxylin and eosin, H&E) from the tissue blocks were reviewed by an experienced pathologist (M.B.) for confirmation of the diagnosis and adequacy of material as well as calculation of the percentage of tumor cells in each case. In 97 cases, the tumor tissue in the paraffin block was adequate for the construction of tissue microarrays (TMA), while for 11 cases all assays were performed on whole tissue sections.

### TMA construction

Tumor tissue specimens were arrayed (3 cores per case, 0.6 mm in diameter) into a recipient paraffin block using a manual arrayer (MTA-1, Beecher Instruments, Sun Prairie, WI, USA). Representative cores from normal (colon, tonsil, placenta, kidney, thyroid, breast, and prostate) and cancerous tissues (melanoma, testicular seminoma, colon carcinoma, squamous head and neck carcinoma, and endometrial carcinoma) were also loaded on the TMA block, serving as positive and negative assay controls.

### Chromogenic in situ hybridization

In order to detect latent EBV infection [6], CISH assays were performed on serial 4 μm-thick sections from the original blocks and the TMA block, using a Bond Max™ autostainer (Leica Microsystems, Wetzlar, Germany), according to the provider’s specifications as described in detail elsewhere [5]. The poly-A probe, used as an indicator of the preservation of mRNA in the cells (positive control), resulted mainly in dark brown nuclear staining and less in a cytoplasmic one.

### Immunohistochemistry – selection of proteins

Immunohistochemical labelling was performed on serial 3 μm-thick sections from the original blocks and the TMA block. Proteins involved in angiogenesis, cell adhesion, assembly, proliferation and differentiation, cell cycle and transcription regulation, DNA repair, microtubule assembly, and inflammation mediation were investigated. A list of the studied proteins, the sources of antigens and the staining procedures for IHC and the cut-offs used [6-11] are quoted in Table 1. The Multi-Cytokeratin (AE1/AE3) was used as a control stain for the identification of tumor cells.

**Table 1:** Studied proteins, primary antibodies, source and dilutions of antibodies, staining patterns, and interpretation (cut-off) criteria used in the present study

**Protein**	**Antibody [clone, source]**	**Antibody dilution**	**Time/Pretreatment**	**Incubation Time**	**Cutoff (%)**	**Staining pattern**
*Cell adhesion molecules*						
E-cadherin	HECD-1^1^	1:30	20 min / ER1	60 min	IRS ≥5	C, C/M
P-cadherin	56^2^	1:200	20 min / ER2	O/N	IRS ≥5	C, C/M
*Cell assembly molecules*						
Fascin-1	IM20^1^	1:400	20 min / ER1	O/N	5%	C
Cytokeratin Multi	AE1/AE3^1^	1:100	10 min / Enzyme 1	20 min	1%	C
*Cell cycle regulators*						
Cyclin D1	SP4^3^	1:80	20 min / ER1	30 min	IA (5%)	N
*Cell growth molecules*						
VEGF-A	VG1^4^	1:75	20 min / ER2	60 min	5%	C
VEGF-C	18-2255, pl^5^	1:250	20 min / ER1	O/N	5%	C
Phospho-mTOR^Ser2448^	49 F9^6^	1:30	20 min / ER1	20 min	5%	C, C/PN
*Cell proliferation molecules*						
Ki67	MIB1^4^	1:70	15 min / ER2	20 min	IA (13%)	N
*Cell receptors*						
EGFR	31 G7^5^	1:50	8 min / Enzyme 2	20 min	10%	M, C/M
VEGFR-2	55B11^6^	1:450	20 min / ER2	O/N	5%	C, C/N
VEGFR-3	KLT9^1^	1:50	15 min / ER1	O/N	5%	C
*DNA repair molecules*						
ERCC1	8 F1^2^	1:300	15 min / ER2	30 min	50%	N, C/N
p53	DO-7^4^	1:100	20 min / ER1	20 min	50%	N, N/C
*Inflammation mediators*						
COX-2	4H12^1^	1:300	20 min / ER1	O/N	IRS≥3	C
*Microtubule assembly*						
MAPT (Tau)	2B2.100^7^	1:100	20 min / ER1	O/N	5%	C, N, C/N
*Molecules with kinase activity*						
Phospho-AKT^Ser473^	D9E^6^	1:150	20 min / ER2	O/N	5%	C, N, C/N
Phospho-GSK-3β^Ser9^	5B3^6^	1:50	20 min / ER1	O/N	5%	C, N, C/N
Phospho-p44/42 MAPK^Thr202/Tyr204^	20 G11^6^	1:1000	20 min/ ER1	O/N	5%	C, N, C/N
*Transcriptional activator or repressor*						
p63	4A4^1^	1:50	20 min / ER2	60 min	5%	N
*Tumor suppressor*						
PTEN	6H2.1^4^	1:300	20 min / ER2	60 min	10%	C, N, C/N

^1^Leica Biosystems, Newcastle, UK; ^2^Thermo Fisher Scientific, Fremont, CA;^3^Spring Bioscience, Pleasanton, CA; ^4^Dako, Glostrup, Denmark; ^5^Invitrogen, Carlsbad, CA; ^6^CST: Cell Signaling Technology, Danvers, MA; ^7^United States Biological, Swampscott, MA.

ER1: Epitope Retrieval 1 (citric acid, pH 6); ER2: EDTA (pH 8.8); IA: image analysis; IRS: immunoreactive score=Intensity (0–3) +% of stained cells (0:0-5%, 1:6-25%, 2:26-50%, 3:>50%); min: minutes; M: membrane; N: nuclear; O/N: overnight; pl: polyclonal; PN: perinuclear; C: cytoplasm; C/M: cytoplasm/membrane.

### Evaluation of the CISH and IHC

The evaluation of CISH and IHC stained sections was done simultaneously by two pathologists (G.K. and M.B.) blinded as to the patients’ clinical characteristics and survival data, according to the criteria quoted in Table 1. Tumor cells containing EBER transcripts were evaluated as positive when an intense, brown, predominantly nuclear staining was present in >1% of tumor cells [12]. Only poly-A probe positive cases were evaluable for EBER status. IHC cut-off values were defined prospectively, based on distributional analyses in frequency histograms and the medical literature. In order not to miss cut-off values with prognostic utility, a Receiver-Operating Curve analysis of IHC staining scores was followed with disease progression as the indicator parameter; this analysis failed to show any cut-off with significant sensitivity and specificity (Table 1). Based on the selected IHC cut-offs, protein expression status was defined for each tumor as either negative or positive, except for Ki67 which was graded as low (≤13%) or high (>13%). Representative cases of CISH and IHC staining are presented in Additional file 1: Figure S1.

### Image analysis

Stained sections for Cyclin D1 and Ki67 were evaluated using an image analysis system as described [5]. The cut-offs used for the evaluation of Cyclin D1 and Ki67 staining are quoted in Table 1.

### Statistical analysis

Categorical data are displayed as frequencies and corresponding percentages, while continuous data by median and range. For the comparison of continuous data the non-parametric Kruskall Wallis test was used, while the comparison of categorical data between groups was performed by Fisher’s exact or Pearson chi-square tests, where appropriate. Overall survival (OS) was measured from the date of treatment randomization to patient’s death or last contact. Progression-free survival (PFS) was measured from the date of treatment randomization to documented disease progression, death without prior documented progression or last contact. Time-to-event distributions were presented using Kaplan-Meier curves and compared using the log-rank test. Unsupervised hierarchical clustering analysis using the majority of the examined biomarkers was conducted. Univariate Cox regression analyses were performed for OS and PFS, to assess the prognostic or predictive significance of biomarkers adjusted for treatment. A backward selection procedure with a removal criterion of p>0.10 was performed in the multivariate Cox regression analysis in order to identify significant factors among examined biomarkers, basic clinicopathological characteristics (categorized as given in Additional file 2: Table S1) and treatment group. For all comparisons the level of significance was set at α=0.05. All results are presented according to reporting recommendations for tumor marker prognostic studies [13]. Analyses were performed with the use of the SPSS v. 17.0 (SPSS, Inc., Chicago, IL), JMP version 8 and SAS version 9.3 (SAS, Institute Inc., Cary, NC, USA).

## Results

### Patient and tumor characteristics – survival times

**Figure 2:**
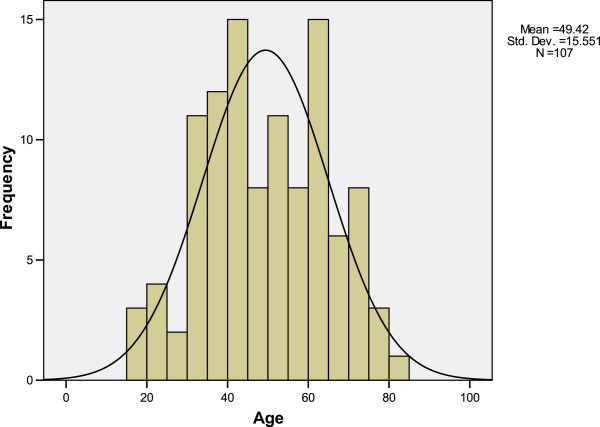
Age distribution frequencies of the studied nasopharyngeal patient population.

Selected patient and tumor characteristics are quoted in Table 2. The male/female ratio was 2.24/1. The majority of patients were of PS 1 or 2, early T stage and advanced lymphnode involvement. Findings deserving closer attention are the normal pattern of age distribution (Figure 2) and the relatively low incidence of WHO Type I disease, given that the population was Caucasian (9.3%).

**Table 2:** Patient demographics and disease characteristics

**Age (years)**				
Median (range)	49 (15–82)			
**Gender**	**N**	**%**		
Men	74	69.2		
Women	33	30.8		
**Performance status**				
0	38	35.5		
1-2	69	64.5		
**T classification**				
T1	23	21.5		
T2	40	37.4		
T3	16	14.9		
T4	28	26.2		
T1+T2	63	58.9		
T3+T4	44	41.1		
**N classification**				
N0	15	14.0		
N1	27	25.2		
N2	44	41.1		
N3a	4	3.7		
N3b	17	15.9		
N0+N1	42	39.3		
N2+N3	65	60.7		
**Clinical stage**				
II-III	64	59.8		
IV	43	40.2		
**WHO classification**				
Type I	10	9.3		
Type II	21	19.6		
Type III	76	71.0		
**History of smoking**				
No	71	66.4		
Yes	36	33.6		
**Alcohol abuse**				
No	100	93.5		
Yes	7	6.5		
**Hemoglobin level**				
≥12	10	9.3		
<12	94	87.9		
Unknown	3	2.8		
**Best Response**	**Group A**		**Group B**	
CR	42	58	38	55
PR	18	25	21	30
ORR	60	83	59	85
SD	3	4	2	3
PD	2	3	5	7
NE	7	10	3	4

CR: complete response; PR: partial response; ORR: overall response rate; SD: stable disease; PD: progressive disease; NE: not evaluated.

The patients have been followed for an additional period of 21.8 months compared to the respective clinical study [5]. After a median follow-up time of 76.8 months (range 42.3-99.2 months), 3-year PFS and OS rate for all patients in the study were 60.7% and 66.4%, respectively. Mean PFS and OS for all patients were 49.7 months and 52.9 months, respectively. No statistically significant differences emerged when time-to-event times were analyzed with respect to the treatment group (mean PFS: 51.1 months for Group A vs. 47.4 months for Group B, p = 0.65; mean OS: 53.4 months for Group A vs. 51.7 months for Group B, p = 0.938).

### Frequency of IHC marker expression

Biomarkers with the most frequent expression (>90% positive cases) were: p53, COX-2, P-cadherin, EBER, phospho-GSK-3β (p-GSK-3β), and Fascin-1. On the contrary, the least IHC-expressed proteins (<50% positive cases) were as follows: VEGFR-2, phospho-mTOR^Ser2448^ (p-mTOR), VEGF-A, and p16 (Table 3).

**Table 3:** Frequency of immunohistochemical expression of the examined biomarkers in nasopharyngeal carcinoma (sorted from the most frequently expressed to the least frequently ones)

**Biomarker**	**No of Cases**	**Positive (%)**	**Negative (%)**
Multi-Cytokeratin	103	102 (99)	1 (1)
COX-2	94	90 (95.7)	4 (4.3)
P-cadherin	97	92 (94.8)	5 (5.2)
EBER	101	94 (93.1)	7 (6.9)
Phospho-GSK-3β^Ser9^	100	92 (92)	8 (8)
Fascin-1	96	88 (91.7)	8 (8.3)
E-cadherin	94	83 (88.3)	11 (11.7)
p63	94	83 (88.3)	11 (11.7)
EGFR	95	83 (87.4)	12 (12.6)
p53	95	82 (86.3)	13 (13.7)
Phospho-AKT^Ser473^	96	79 (82.3)	17 (17.7)
Ki67	100	78 (78)	22 (22)
ERCC1	105	78 (74.3)	27 (25.7)
MAPT (Tau)	104	74 (71.2)	30 (28.8)
VEGF-C	100	64 (64)	36 (36)
Phospho-p44/42 MAPK^Thr202/Tyr204^	95	60 (63.2)	35 (36.8)
PTEN	102	64 (62.7)	38 (37.3)
VEGFR-3	101	60 (59.4)	41 (40.6)
Cyclin D1	102	53 (52)	49 (48)
VEGFR-2	95	44 (46.3)	51 (53.7)
Phospho-mTOR^Ser2448^	101	38 (37.6)	63 (62.4)
VEGF-A	100	36 (36)	64 (64)
p16	105	5 (4.8)	100 (95.2)

### Association of protein expression with clinicopathological characteristics and response to treatment

The statistically significant correlations of patients’ clinicopathological characteristics with the IHC proteins expression are presented in Additional file 2: Table S1. Notably, PTEN and p63 expression patterns were clearly linked with favorable characteristics: patient PS and T stage (for the former), T and AJCC TNM stage (for the latter). Positive phospho-AKT (p-AKT) and phospho-p44/42MAPK (p-MAPK) expression were related to male gender, positive p-GSK-3β and ERCC1 expression depicted a more advanced age at diagnosis, and deficiency of ERCC1 and p-mTOR expression were correlated to poor patient PS. As expected, positive EBER expression status was linked to WHO Type III histology. Unexpectedly, Ki67-positive tumors were more frequently associated with earlier N stage and good PS; likewise, Cyclin D1 expression was more often positive in tumors of earlier AJCC TNM stage.

Associations between the expression status of the tested biomarkers and response to treatment did not reveal significant predictive values for any of them (Data not shown).

### Paired associations of protein expression as detected by IHC

Additional file 3: Table S2 presents all statistically significant paired associations of protein IHC expression. The most interesting paired interactions were as follows: p63/p-AKT, p63/Ki67, p-AKT/Ki67, p-AKT/ERCC1, Ki67/ERCC1, Ki67/PTEN, Ki67/Cyclin D1, p-mTOR/ERCC1, and p-mTOR/VEGFR3.

Consequently, the most informative biomarkers in NPC with regards to IHC expression were: p63, p-AKT, Ki67, ERCC1, CyclinD1, p53, COX-2, and p-mTOR.

### Profiling cluster models of protein expression

In an effort to study the complex interactions among the examined biomarkers, cluster analysis of the IHC protein expression was performed.

**Figure 3:**
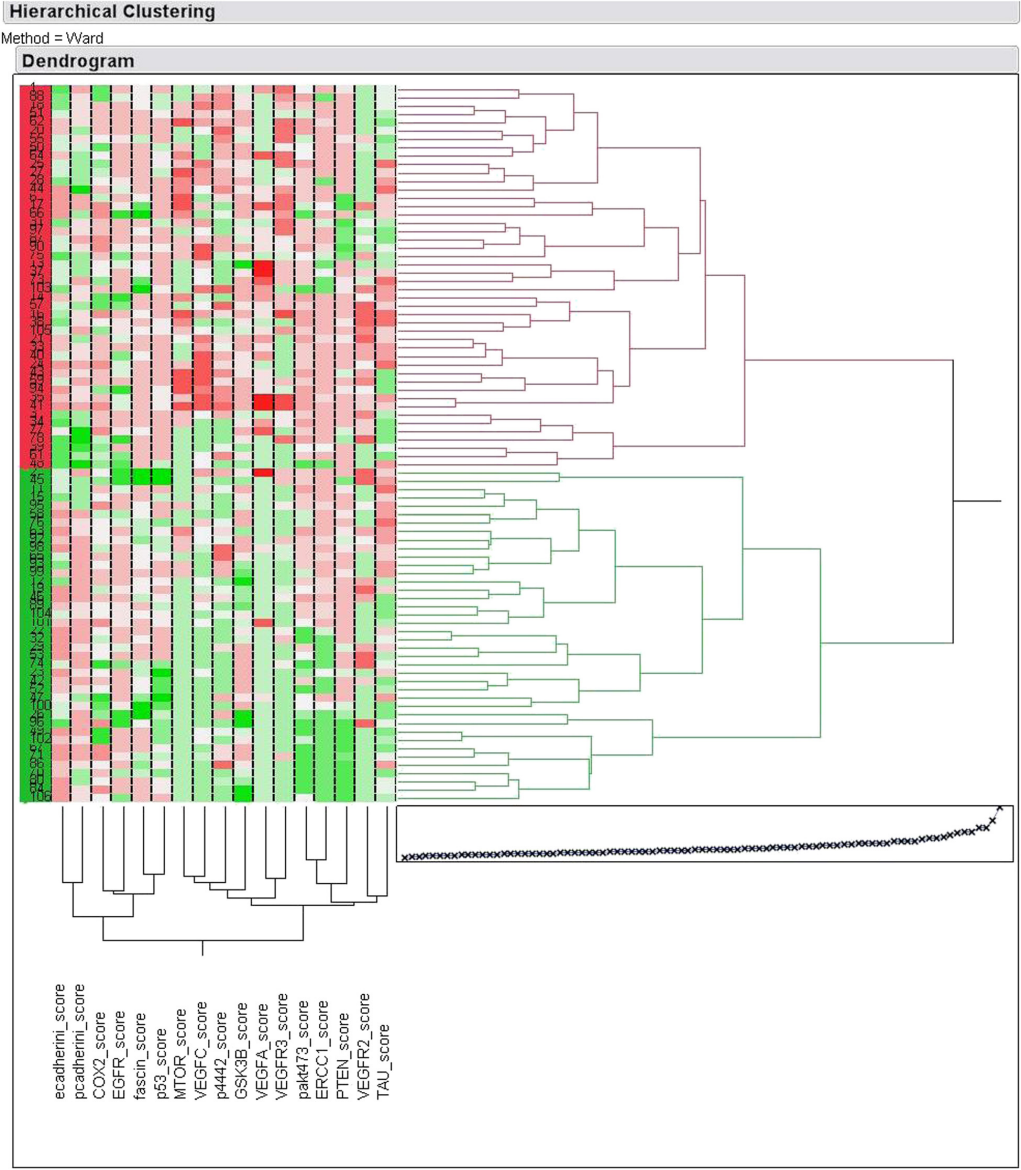
**Hierarchical clustering analysis of the studied biomolecules in nasopharyngeal carcinoma.** Red and green signals indicate cases with increased and decreased protein expression, respectively; white signals indicate missing cases. Two clusters are distinguished, red and green, with different predominant patterns of protein expression.

**Figure 4:**
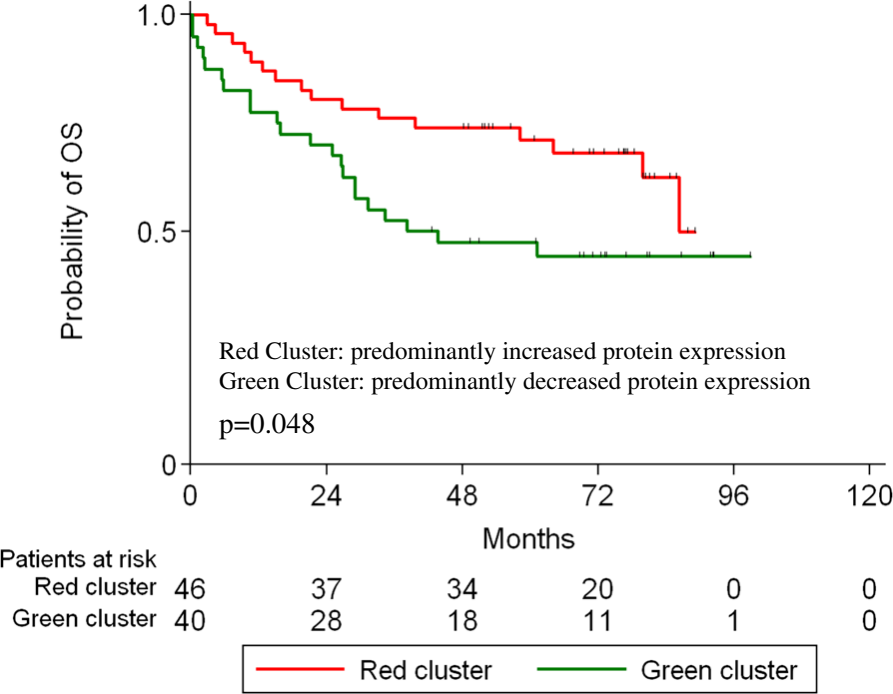
Prognostic significance for overall survival of the red and green clusters, with predominantly increased and decreased protein expression, respectively.

Firstly, all protein targets were included in the analysis and gradually increasing numbers of clusters were tested for their prognostic significance with respect to PFS and OS as well as their association with T and N status, AJCC TNM stage, and overall response rate (ORR) to treatment. The only model with prognostic significance was a 2-cluster one, with a borderline association to favorable OS (p=0.048) (Figures 3 and 4) and earlier TNM stage (p=0.027 for stage IIB-III vs. IV). Cluster analysis for each of the two treatment groups (A and B) did not result in the emergence of any meaningful prognostic significance.

A following step was to look at a more compact set of protein targets (such as p-AKT, p-mTOR, PTEN, VEGF-A, ERCC1, and p53) based on their relative significance in driving carcinogenesis or their possible synergistic action. In this case, no cluster appeared to confer prognostic information for any of the endpoints tested.

### Univariate analysis

In univariate Cox regression analysis adjusted for treatment, the only biomarkers with positive prognostic value were EBER and p63, the latter being important also in the premature translational analysis of the relevant clinical study [7]. Particularly, EBER expression was linked to a favorable OS in a statistically significant way (hazard ratio HR = 0.38, 95% CI = 0.15-0.99, p = 0.048), while its association to improved PFS was only of borderline significance (HR = 0.44, 95% CI = 0.17-1.14, p = 0.09). The favorable prognostic value of positive p63 expression was borderline only for OS (HR = 0.48, 95% CI = 0.21-1.09, p=0.08) (Table 4 and Additional file 4: Figures S2).

**Table 4:** Univariate Cox regression analysis for the examined biomarkers adjusted for treatment

	**OS**			**PFS**		
	**HR**	**95% CI**	**Wald’s p**	**HR**	**95% CI**	**Wald’s p**
**EBER**						
Negative	1			1		
Positive	0.38	0.15-0.99	**0.048**	0.44	0.17-1.14	**0.09**
**p63**						
Negative	1			1		
Positive	0.48	0.21-1.09	**0.08**	0.56	0.25-1.28	0.17
**Cyclin D1**						
Negative						
Group B vs. Group A	0.44	0.18-1.04	**0.062**	0.5	0.22-1.16	0.11
Positive						
Group B vs. Group A	2.02	0.81-5.07	0.13	2.64	1.08-6.48	**0.034**

**Figure 5:**
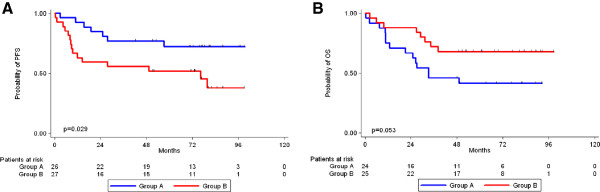
**Prognostic significance of Cyclin D1 expression.** Log-Rank test for the effect of Cyclin D1 expression on progression-free and overall survival, stratified by treatment group (Group A: patients treated with induction chemotherapy followed by concurrent chemo-radiotherapy; Group B: patients treated with concurrent chemo-radiotherapy only); (**A**) positive and (**B**) negative Cyclin D1 expression.

Of special interest was the predictive significance of Cyclin D1 expression, which emerged only when the patient population was adjusted for the administered treatment (Group A vs. Group B). In this case, immunopositivity for Cyclin D1 resulted in a statistically significant worse prognosis in terms of PFS for patients in Group B (HR = 2.64, 95% CI = 1.08-6.48, p=0.034) (Table 4), while the absence of Cyclin D1 expression conferred a trend of better OS for the same Group (HR = 0.44, 95% CI = 0.18-1.04, p=0.062) (Table 4). Log-Rank test was subsequently performed, which confirmed the results of Cox regression analysis mentioned above (Figures 5A and 5B).

### Multivariate analysis

The following parameters were pointed out by multivariate analysis as the significant prognostic indicators for both PFS and OS: age at diagnosis, treatment group, PS, AJCC TNM stage, p-mTOR and ERCC1 expression. Notably, not only the same variables were indicated as important for both PFS and OS, but also the magnitude of significance (denoted by HR value) was similar in both conditions (Tables 5 and 6).

**Table 5:** Multivariate Cox model for progression-free survival

	**Parameter Estimate**	**Standard Error**	**p-value**	**HR**	**95% CI**
**Age**	0.042	0.011	<0.001	1.043	1.020-1.065
**Group**					
Group B vs. Group A	0.41	0.33	0.21	1.5	0.89-2.84
**PS**					
1-2 vs. 0	1.5	0.41	<0.001	4.47	1.98-10.01
**Stage**					
IV vs. II-III	0.95	0.33	0.005	2.58	1.34-4.96
**p-mTOR**					
Positive vs. Negative	−0.8	0.33	0.031	0.45	0.22-0.93
**ERCC1**					
Positive vs. Negative	1.02	0.41	0.013	2.79	1.24-6.29

**Table 6:** Multivariate Cox model for overall survival

	**Parameter Estimate**	**Standard Error**	**p-value**	**HR**	**95% CI**
**Age**	0.047	0.011	<0.001	1.048	1.024-1.073
**Group**					
Group B vs. Group A	0.23	0.33	0.5	1.25	0.66-2.40
**PS**					
1-2 vs. 0	1.4	0.42	<0.001	4.06	1.79-9.19
**Stage**					
IV vs. II-III	0.92	0.34	0.007	2.5	1.28-4.89
**p-mTOR**					
Positive vs. Negative	−0.76	0.38	0.044	0.47	0.22-0.98
**ERCC1**					
Positive vs. Negative	0.93	0.42	0.028	2.54	1.11-5.81

Particularly, advanced age, the addition of induction chemotherapy, poor PS, AJCC stage IV, and positive ERCC1 expression were associated with a higher probability of disease progression and death. On the contrary, positive p-mTOR expression was related to a decreased risk of progression and death (Additional file 4: Figures S2).

Compared to the preliminary analyses performed in the context of the respective clinical study [7], Ki67 and p63 lost their independent prognostic value in the longer follow-up, in contrast to age, PS, and AJCC TNM stage which maintained their significance.

## Discussion

In contrast to the progress seen in the molecular classification, pathogenesis elucidation and management of quite a few neoplasms, little has been achieved towards personalized approach in the NPC field so far. In contrast to the paradigm of other neoplasms, no activating mutations, such as EGFR or PI3K, are known to drive nasopharyngeal carcinogenesis [14,15]. It seems that nasopharyngeal carcinogenesis is far more composite than the almost ubiquitous EBV presence depicts. As a result, survival rates of patients with advanced disease remain poor, especially for non-endemic populations [16]. During the last few years, we have witnessed combined efforts to unravel NPC pathogenesis by utilization of multiple gene expression profile datasets, definition of novel gene signatures and molecular or cytokine markers, integration of miRNA technologies, and exploration of epigenetics.

The present study incorporates novel features which differentiate it from the rest utilizing IHC in NPC. In particular, 21 protein targets have been simultaneously profiled for their expression levels in nasopharyngeal tumors, in order to observe possible cross-talks between pathways. Biomarkers were carefully chosen, in order to include representatives from as many as possible oncogenic processes, as reviewed recently [17]. In view of this, the clustering analysis performed is considered even more informative. More importantly, the study population was a Caucasian one, representing not only the entire Greek region but also the Balkan Peninsula, since HECOG-affiliated hospitals from both Greece and Romania have participated in this study. Although a considerable number of Caucasian NPC patients have been studied during the last decades [18-23], the respective series have either focused just on clinical parameters or have investigated only a limited number of biomarkers.

The high frequency of EBER CISH expression was not surprising, even for the Caucasian population of our study, as EBV infection is almost ubiquitous globally and plays a major role in the pathogenesis of NPC in both endemic and non-endemic areas [1]. Generally, our study confirmed that the well-established characteristics of EBV-related NPC are also applicable to Caucasian NPC patients. Namely, the strong connection of EBV to WHO Type II and III histology as well as to the more favorable disease course [24]. Importantly, the univariate analysis indicated EBER expression as a significant prognostic factor of improved OS.

Cyclin D1 is one of the key proteins involved in cell cycle control, a process considered as critical in the development of NPC [25]. Although our study did not find as high uniform Cyclin D1 expression as reported in the literature [7], IHC expression levels were interestingly informative. Firstly, a statistically significant association between Cyclin D1 and Ki67 as well as between Cyclin D1 and p-AKT expression was indicated, pointing to an active cell proliferation axis, which has already been investigated as a therapeutic target in NPC [26]. More importantly, univariate analysis pointed to a predictive role for Cyclin D1 protein levels, as differences in PFS and OS emerged only when the study population was adjusted for treatment. In particular, patients treated with induction chemotherapy prior to chemo-radiotherapy fared significantly better (in terms of PFS) in the case of positive Cyclin D1 expression, while in the absence of Cyclin D1 this finding was inverted (for OS). Consequently, it seems that tumors with enhanced Cyclin D1 function are vulnerable to more aggressive treatment. This result is in fine agreement with the reported radio-sensitivity of Cyclin-enriched nasopharyngeal [7], laryngeal [27], and breast [28] cancers.

The function of p63, a transcription factor and member of the p53 family, is rather complicated in NPC, since two major classes of p63 isoforms have been identified: the full-length TAp63 and the N-terminal truncated ΔNp63 [29]. The two isoforms exert opposite purposes; TAp63 isoform has similar function to the wild-type p53 protein, while ΔNp63 is thought to antagonize TAp63 and p53 in target gene regulation. Each isoform can be targeted by a specific antibody for IHC evaluation, while a pan-p63 one binds to common epitopes. Consequently, reports of overexpression and oncogenic properties of p63 in NPC [30,31] should be construed through this prism, also bearing in mind that the predominant isoform is the truncated ΔNp63 one. In our study, a pan-p63 antibody was utilized, thus limiting the importance of the borderline favorable prognostic value for OS, which emerged for p63 in the univariate analysis. Accordingly, the strong correlation of p63 expression with p-AKT and Ki67 as well as its association with early disease stage should be interpreted with caution.

The virtual absence of p16 IHC expression in our study (only 5% positive tumors) has been constitutively documented in nasopharyngeal cancers [32], attributable mainly to aberrant methylation or even to gene deletion. In contrast to reports supporting a predictive role of p16 to both chemotherapy (5-flouoruracil and cisplatin) [33] and radiotherapy [34], our study did not find any connection of p16 protein expression with disease outcome. Likewise, despite the documented unfavorable value of p16 expression in NPC [7,35], such a role did not emerge in our study. The inevitable small number of p16-positive cases undoubtedly calls for a cautious reading of these results.

Activation of the PI3K/AKT/mTOR pathway has been reported in NPC several years ago [36]. AKT phosphorylation can be either a result of LMP1 [37] or EGFR [14] action on PI3K or a compensatory consequence of decreased PTEN levels [38]. However, EBER/p-AKT or EGFR/p-AKT protein interactions did not emerge in our study. Moreover, the comparable expression levels of p-AKT and PTEN argue in favor of a downward activation of AKT instead of a compensatory one. p-AKT association with the Ki67 status may reflect the known propensity of AKT-activated NPC to metastasize [39]; however, we could not confirm the contribution of decreased E-cadherin levels to this phenomenon [40].

Cadherin IHC expression did not acquire any significance in our study with regard to disease prognosis or association with the rest biomolecules examined. Despite the reported depletion of E-Cadherin expression in NPC tissues in comparison to the normal ones [32,41], a high level of IHC expression was observed in our study, both for P- and for E-Cadherin. One possible explanation is the low IHC cut-off that was selected, compared to other reports [42]. On the other hand, the enhanced Cadherin expression might not merely be an artifact but could reflect a lack of its contribution to NPC pathogenesis in Caucasian patients. Such a scenario would justify the absence of any unfavorable prognostic value, contrary to reports of low tumor Cadherin levels in NPC patients with advanced disease stage and decreased survival [43].

The predictive role of high ERRC1 protein levels to cisplatin-containing regimens has been firstly described in ovarian cancer 20 years ago [44]. However, it was only recently that similar reports were published in NPC [45]. In line with this evidence, multivariate analysis in our study showed a significant unfavorable association of increased ERCC1 expression with both PFS and OS. Since cisplatin was the chemotherapy backbone in both patient groups, this outcome could be explained as a negative predictive effect of high ERCC1 protein levels. In addition, a poor prognostic value of ERCC1 could also be contemplated, as increased ERCC1 protein levels were significantly associated with aggressive disease characteristics, such as positive Ki67 and p-AKT IHC expression.

Loss of PTEN expression is a frequent event in NPC [46], accounting for the extensive deregulation of cellular signaling pathways (e.g. PI3K/AKT/mTOR and Wnt) and the metastatic propensity via induction of the epithelial-mesenchymal transition phenotype. Our study, in line with the literature evidence, has found a relatively low rate of PTEN expression. Moreover, PTEN presence was significantly associated with early tumor stage, a finding which has been also described in Chinese NPC patients [47].

Increased COX-2 IHC expression in NPC has been linked to the presence of lymph node metastasis [48] and worse survival, as well to enhanced sensitivity to the radiotherapy effects [49]. In contrast to such well-established evidence, no prognostic or predictive role for COX-2 was revealed in our study, despite the higher COX-2 expression frequency compared to previous reports [50]. However, in line with our observations are a few studies [51] which indicate no prognostic significance of COX-2 or even a positive one [52], thus perplexing COX-2 contribution to NPC pathogenesis. Commonly reported co-expression patterns, such as COX-2/EGFR/VEGF [53], COX-2/EGFR [54], and COX-2/LMP1 [55] were not confirmed in the present study. The axis LMP1/COX-2/VEGF [56], in which COX-2 is promoted by LMP1 and subsequently induces VEGF, was also not manifested in our Caucasian patient series.

However extensive our investigation was, a bunch of biomolecules and processes were not shown to contribute in any way to NPC pathogenesis, despite the strong literature evidence to the contrary. In particular, p-MAPK was neither evident in our tumor series nor was clinically significant, counter to reports of an activated and tumor-promoting MAPK in NPC [57]. Likewise, despite p-GSK-3β being reported as a vital member of the PI3K/AKT/GSK-3β/Cyclin D1 pathway in NPC promotion [26,36,46], the present study did not result in such evidence. Similarly, p53 expression was not informative in terms of disease prognosis or interaction with any other biomarker. Several reports state the overexpression or accumulation of p53 protein in the vast majority of NPC [58] and a resulting tumor-promoting role ; however, p53 expression was not detected to that extent in our study. Anyway, the functional status of p53 is disputable in NPC [59], which could explain the absence of its prognostic value [59], as indicated also in our study. The antagonizing effect of ΔNp63 protein on p53, as quoted above, renders the conclusion-making procedure even more insecure. Fascin, an actin cross-linking protein, has been implicated in the progression of various neoplasms, including NPC, mainly through the promotion of cell migration and adhesion [60]. In spite of Fascin’s high expression frequency in our study, any significant correlation of Fascin-1 to NPC pathogenesis cannot be implied.

The absence of any prognostic role for EGFR in our Caucasian population, despite its high expression frequency, is a result which needs particular consideration. There is a substantial bulk of evidence concerning the unfavorable prognostic value of EGFR both for disease stage and for outcome [61], even in Caucasian populations [62], the only exception being a recent Korean study [51]. Additionally, several co-expression patterns have been described, such as EGFR/COX-2 [53,54], EGFR/VEGF [53], and EGFR/AKT [14], not being manifested in our series. Although EBV presence is believed to induce EGFR [63], such a paired expression model was also not found. The paucity of EGFR-expression information in the present study could be interpreted in the context of the evolving understanding of EGFR role in NPC pathogenesis; EGFR downstream signaling molecules are numerous in NPC and are still being defined [64]. The relative inefficacy of EGFR-targeting attempts in NPC [65], in contrast to HNSCC [66], serves as a reminder of the underlying complexity.

VEGF-A and -C expression status were also not informative with regard to prognosis or any association with clinicopathological variables. In spite of the abundant literature references of an adverse prognostic role of VEGF expression in NPC [67,68] and VEGF-C in particular [69], no such effect was shown. Furthermore, several co-expression pairs have been repeatedly described, mainly between VEGF, COX-2, EGFR, and LMP1 [56,70], which were not prominent in our tumor series. Anyway, the exact setting in which angiogenesis exerts its tumor-promoting action needs to be clarified, as depicted by the modest results of anti-angiogenic therapy so far [71].

Phosphorylated-mTOR expression emerged as an independent favorable prognostic factor in multivariate analysis, although with borderline significance. mTOR is linked with a promoting role in nasopharyngeal carcinogenesis [72] and a unanimous adverse prognostic value in NPC [73], which is alleviated by mTOR inhibition [38]; thus, the aforementioned result is considered as unexpected. Possible hypotheses for this discrepancy are: a) the presence of p53 expression in all p-mTOR tumors could account for a favorable outcome upon treatment and b) multiple comparisons in a sample underpowered for such exhausting statistical analyses could lead to an artifact. Similarly, the association of increased Ki67 with an earlier N stage and good PS is rather surprising and could be attributed either to a statistical artifact, as mentioned above, or to the IHC cut-off level which have been used.

Clustering analysis did not reveal any discriminator group of genes/biomolecules which could portray a specific driving scenario of carcinogenesis in NPC. Moreover, no NPC subtype emerged on the basis of differential protein expression, which could point towards potential therapeutic targets. Following the breast cancer paradigm [74], the reported gene-profiling approaches in NPC end up to different gene sets [75,76], calling once again for the need of bioinformatics implementation to data interpretation.

It should be noted that the reported high frequency of WHO Type I NPC histology in Caucasian populations (approximately 25%) [2] was not represented in our study (only 9%). Possibly, Balkan NPC patients represent an intermediate population in terms of epidemiology, as Balkan Peninsula shares the Mediterranean Basin and neighbors to North Africa, which is a known endemic NPC area.

The results of the present study should be interpreted on the notion of the inherent limitations of IHC. Protein expression levels often mirror the mechanisms leading to cellular growth deregulation; however, they have to be translated along with the coexisting genetic and epigenetic alterations. Moreover, clustering analysis is unable to provide a perfect outline of the complex interactions between the tumor-promoting networks; small-size samples and multiple comparisons undermine the validity of the results. Last but not least, since several phospho-antibodies were utilized, consideration should be paid to the disputable reliability of FFPE IHC for phosphorylated epitopes [77].

## Conclusions

Despite the existence of published evidence to the contrary, the extensive IHC study and profiling of a broad variety of biomolecules did not result in tangible conclusions for many of them. However, our study came to conclusions which could represent a step forward in disclosing the pathogenesis of NPC in Caucasian populations. The favorable prognostic value of EBER and p63, the treatment-dependent prognostic significance of Cyclin D1, as well as the independent prognostic value of ERCC1 and p-mTOR protein levels could be pointed as the most interesting ones. What lies ahead is the prospective validation of these biomarkers and, hopefully, a meaningful benefit for NPC patients.

## Abbreviations

NPC: Nasopharyngeal carcinoma; EBV: Epstein-Barr virus; HNSCC: Head and neck squamous cell carcinomas; IHC: Immunohistochemistry; LA-NPC: Locally advanced NPC; CRT: Chemo-radiotherapy; HeCOG: Hellenic Cooperative Oncology Group; FFPE: Formalin-fixed paraffin-embedded; EBER: EBV-related small RNA; CISH: Chromogenic in situ hybridization; H&E: Hematoxylin and eosin; TMA: Tissue microarrays; PFS: Progression-free survival; OS: Overall survival; ORR: Overall response rate; HR: Hazard ratio; PS: Performance status; ECOG: Eastern Cooperative Oncology Group.

## Competing interests

The authors declare that they have no competing interests.

## Authors’ contributions

DK analyzed the data and drafted the manuscript. MB and GK assessed the IHC and helped to draft the manuscript. LR, DA and VV participated in the design and coordination of the study and analyzed the data. SC participated in the design of the study and helped to draft the manuscript. ES participated in the design and coordination and helped to draft the manuscript. EC participated in the design and coordination of the study. GF conceived of the study and participated in its design and coordination. All authors read and approved the final manuscript.

## Pre-publication history

The pre-publication history for this paper can be accessed here:

http://www.biomedcentral.com/1472-6890/13/1/prepub

## Supplementary Material

Additional file 1 **Figure S1.** Protein expression detected by IHC and CISH in tissue microarrays from nasopharyngeal carcinoma cases. (**a**) p53; (**b**) EGFR; (**c**) COX-2; (**d**) VEGF-A; (**e**) MAPT; (**f**) E-cadherin; (**g**) PTEN; (**h**) p-GSK-3β; (**i**) VEGF-C; (**j**) ERCC1; (**k**) Fascin-1; (**l**) p-AKT; (**m**) p-p44/42 MAPK; (**n**) VEGFR-2; (**o**) Cyclin D1; (**p**) P-cadherin; (**q**) p-mTOR; (**r**) p63; (**s**) VEGFR-3; (**t**) Ki67; (**u**) Multi-Cytokeratin; (**v**) mRNA probe (CISH); (**w**) EBER probe (CISH). Original magnification ×100. Click here for file

Additional file 2 **Table S1.** Statistical correlations of biomolecules expression with clinicopathological parameters. Click here for file

Additional file 3 ** Table S2.** Paired associations of protein expression (Fisher’s exact test). Click here for file

Additional file 4 **Figure S2.** Prognostic significance of EBER, p63, mTOR, ERCC1 and Cyclin D1 protein expression for progression-free and overall survival (Log-Rank test). For Cyclin D1, all patients have been included, irrespective of the treatment administered. Click here for file

## References

[B1] Chang ET Adami HO The enigmatic epidemiology of nasopharyngeal carcinoma Cancer Epidemiol Biomarkers Prev 2006 15 10 1765 1777 10.1158/1055-9965.EPI-06-0353 17035381

[B2] Curado MP Edwards B Shin H Storm H Ferlay J Heanue M Boyle P Cancer Incidence in Five Continents 2007 Lyon, France: IARC Scientific Publications

[B3] Dancey JE Bedard PL Onetto N Hudson TJ The genetic basis for cancer treatment decisions Cell 2012 148 3 409 420 10.1016/j.cell.2012.01.014 22304912

[B4] Lee AW Sze WM Au JS Leung SF Leung TW Chua DT Zee BC Law SC Teo PM Tung SY Treatment results for nasopharyngeal carcinoma in the modern era: the Hong Kong experience Int J Radiat Oncol Biol Phys 2005 61 4 1107 1116 10.1016/j.ijrobp.2004.07.702 15752890

[B5] Fountzilas G Ciuleanu E Bobos M Kalogera-Fountzila A Eleftheraki AG Karayannopoulou G Zaramboukas T Nikolaou A Markou K Resiga L Induction chemotherapy followed by concomitant radiotherapy and weekly cisplatin versus the same concomitant chemoradiotherapy in patients with nasopharyngeal carcinoma: a randomized phase II study conducted by the Hellenic Cooperative Oncology Group (HeCOG) with biomarker evaluation Ann Oncol 2012 23 2 427 435 10.1093/annonc/mdr116 21525406

[B6] Chang KL Chen YY Shibata D Weiss LM Description of an in situ hybridization methodology for detection of epstein-barr virus RNA in paraffin-embedded tissues, with a survey of normal and neoplastic tissues Diagn Mol Pathol 1992 1 4 246 255 1342973

[B7] Hwang CF Cho CL Huang CC Wang JS Shih YL Su CY Chang HW Loss of cyclin D1 and p16 expression correlates with local recurrence in nasopharyngeal carcinoma following radiotherapy Ann Oncol 2002 13 8 1246 1251 10.1093/annonc/mdf215 12181248

[B8] Cheang MC Chia SK Voduc D Gao D Leung S Snider J Watson M Davies S Bernard PS Parker JS Ki67 index, HER2 status, and prognosis of patients with luminal B breast cancer J Natl Cancer Inst 2009 101 10 736 750 10.1093/jnci/djp082 19436038 PMC2684553

[B9] Dabbs DJ Sturtz K Zaino RJ The immunohistochemical discrimination of endometrioid adenocarcinomas Hum Pathol 1996 27 2 172 177 10.1016/S0046-8177(96)90371-8 8617459

[B10] Bamias A Karina M Papakostas P Kostopoulos I Bobos M Vourli G Samantas E Christodoulou C Pentheroudakis G Pectasides D A randomized phase III study of adjuvant platinum/docetaxel chemotherapy with or without radiation therapy in patients with gastric cancer Cancer Chemother Pharmacol 2010 65 6 1009 1021 10.1007/s00280-010-1256-6 20130877

[B11] Rakha EA Putti TC Abd El-Rehim DM Paish C Green AR Powe DG Lee AH Robertson JF Ellis IO Morphological and immunophenotypic analysis of breast carcinomas with basal and myoepithelial differentiation J Pathol 2006 208 4 495 506 10.1002/path.1916 16429394

[B12] Fan SQ Ma J Zhou J Xiong W Xiao BY Zhang WL Tan C Li XL Shen SR Zhou M Differential expression of epstein-barr virus-encoded RNA and several tumor-related genes in various types of nasopharyngeal epithelial lesions and nasopharyngeal carcinoma using tissue microarray analysis Hum Pathol 2006 37 5 593 605 10.1016/j.humpath.2006.01.010 16647958

[B13] McShane LM Altman DG Sauerbrei W Taube SE Gion M Clark GM Reporting recommendations for tumor marker prognostic studies J Clin Oncol 2005 23 36 9067 9072 10.1200/JCO.2004.01.0454 16172462

[B14] Yip WK Leong VC Abdullah MA Yusoff S Seow HF Overexpression of phospho-Akt correlates with phosphorylation of EGF receptor, FKHR and BAD in nasopharyngeal carcinoma Oncol Rep 2008 19 2 319 328 18202777

[B15] Lee SC Lim SG Soo R Hsieh WS Guo JY Putti T Tao Q Soong R Goh BC Lack of somatic mutations in EGFR tyrosine kinase domain in hepatocellular and nasopharyngeal carcinoma Pharmacogenet Genomics 2006 16 1 73 74 10.1097/01.fpc.0000184959.82903.02 16344724

[B16] Sun LM Li CI Huang EY Vaughan TL Survival differences by race in nasopharyngeal carcinoma Am J Epidemiol 2007 165 3 271 278 17090616 10.1093/aje/kwk008

[B17] Chou J Lin YC Kim J You L Xu Z He B Jablons DM Nasopharyngeal carcinoma–review of the molecular mechanisms of tumorigenesis Head Neck 2008 30 7 946 963 10.1002/hed.20833 18446839 PMC3046044

[B18] Jiong L Berrino F Coebergh JW Variation in survival for adults with nasopharyngeal cancer in Europe, 1978–1989. EUROCARE Working Group Eur J Cancer 1998 34 14 Spec No 2162 2166 10070282 10.1016/s0959-8049(98)00322-0

[B19] Ertan Y Hekimgil M Karaarslan S Soydan S Expression of epstein-barr-virus-encoded small nuclear RNA in nasopharyngeal carcinomas of aegean Turkish patients Virchows Arch 2008 452 4 411 414 10.1007/s00428-008-0589-6 18299891

[B20] d’Espiney Amaro C Montalvao P Henriques P Magalhaes M Olias J Nasopharyngeal carcinoma: our experience Eur Arch Otorhinolaryngol 2009 266 6 833 838 10.1007/s00405-008-0822-6 18830701

[B21] Terzic TT Boricic MI Pendjer IP Ruzic Zecevic DT Tomanovic NR Brasanac DC Boricic IV Prognostic significance of clinical parameters and epstein-barr virus infection in non-endemic undifferentiated carcinoma of nasopharyngeal type: a serbian report Med Oncol 2011 28 4 1325 1330 10.1007/s12032-010-9551-y 20446059

[B22] Sidler D Thum P Winterhalder R Huber G Haerle SK Undifferentiated carcinoma of nasopharyngeal type (UCNT): a Swiss single-institutional experience during 1990–2005 Swiss Med Wkly 2010 140 19–20 273 279 19950040 10.4414/smw.2010.12844

[B23] Pasini E Caggiari L Dal Maso L Martorelli D Guidoboni M Vaccher E Barzan L Franchin G Gloghini A De Re V Undifferentiated nasopharyngeal carcinoma from a nonendemic area: protective role of HLA allele products presenting conserved EBV epitopes Int J Cancer 2009 125 6 1358 1364 10.1002/ijc.24515 19536817

[B24] Nakao K Mochiki M Nibu K Sugasawa M Uozaki H Analysis of prognostic factors of nasopharyngeal carcinoma: impact of in situ hybridization for epstein-barr virus encoded small RNA 1 Otolaryngol Head Neck Surg 2006 134 4 639 645 10.1016/j.otohns.2005.11.022 16564389

[B25] Hui AB Or YY Takano H Tsang RK To KF Guan XY Sham JS Hung KW Lam CN van Hasselt CA Array-based comparative genomic hybridization analysis identified cyclin D1 as a target oncogene at 11q13.3 in nasopharyngeal carcinoma Cancer Res 2005 65 18 8125 8133 10.1158/0008-5472.CAN-05-0648 16166286

[B26] Ong CS Zhou J Ong CN Shen HM Luteolin induces G1 arrest in human nasopharyngeal carcinoma cells via the Akt-GSK-3beta-Cyclin D1 pathway Cancer Lett 2010 298 2 167 175 10.1016/j.canlet.2010.07.001 20655656

[B27] Yoo SS Carter D Turner BC Sasaki CT Son YH Wilson LD Glazer PM Haffty BG Prognostic significance of cyclin D1 protein levels in early-stage larynx cancer treated with primary radiation Int J Cancer 2000 90 1 22 28 10.1002/(SICI)1097-0215(20000220)90:1<22::AID-IJC3>3.0.CO;2-T 10725854

[B28] Turner BC Gumbs AA Carter D Glazer PM Haffty BG Cyclin D1 expression and early breast cancer recurrence following lumpectomy and radiation Int J Radiat Oncol Biol Phys 2000 47 5 1169 1176 10.1016/S0360-3016(00)00525-3 10889369

[B29] Crook T Nicholls JM Brooks L O’Nions J Allday MJ High level expression of deltaN-p63: a mechanism for the inactivation of p53 in undifferentiated nasopharyngeal carcinoma (NPC)? Oncogene 2000 19 30 3439 3444 10.1038/sj.onc.1203656 10918601

[B30] Fotheringham JA Mazzucca S Raab-Traub N Epstein-Barr virus latent membrane protein-2A-induced DeltaNp63alpha expression is associated with impaired epithelial-cell differentiation Oncogene 2010 29 30 4287 4296 10.1038/onc.2010.175 20498633 PMC2912443

[B31] Chiang CT Chu WK Chow SE Chen JK Overexpression of delta Np63 in a human nasopharyngeal carcinoma cell line downregulates CKIs and enhances cell proliferation J Cell Physiol 2009 219 1 117 122 10.1002/jcp.21656 19089994

[B32] Huang GW Mo WN Kuang GQ Nong HT Wei MY Sunagawa M Kosugi T Expression of p16, nm23-H1, E-cadherin, and CD44 gene products and their significance in nasopharyngeal carcinoma Laryngoscope 2001 111 8 1465 1471 10.1097/00005537-200108000-00025 11568585

[B33] Chow LS Wang X Kwong DL Sham JS Tsao SW Nicholls JM Effect of p16INK4a on chemosensitivity in nasopharyngeal carcinoma cells Int J Oncol 2000 17 1 135 140 10853030

[B34] Lin HS Berry GJ Sun Z Fee WE Jr Cyclin D1 and p16 expression in recurrent nasopharyngeal carcinoma World J Surg Oncol 2006 4 62 10.1186/1477-7819-4-62 16953893 PMC1569377

[B35] Makitie AA MacMillan C Ho J Shi W Lee A O’Sullivan B Payne D Pintilie M Cummings B Waldron J Loss of p16 expression has prognostic significance in human nasopharyngeal carcinoma Clin Cancer Res 2003 9 6 2177 2184 12796384

[B36] Morrison JA Gulley ML Pathmanathan R Raab-Traub N Differential signaling pathways are activated in the Epstein-Barr virus-associated malignancies nasopharyngeal carcinoma and Hodgkin lymphoma Cancer Res 2004 64 15 5251 5260 10.1158/0008-5472.CAN-04-0538 15289331

[B37] Mei YP Zhou JM Wang Y Huang H Deng R Feng GK Zeng YX Zhu XF Silencing of LMP1 induces cell cycle arrest and enhances chemosensitivity through inhibition of AKT signaling pathway in EBV-positive nasopharyngeal carcinoma cells Cell Cycle 2007 6 11 1379 1385 10.4161/cc.6.11.4274 17507800

[B38] Ma BB Lui VW Hui EP Lau CP Ho K Ng MH Cheng SH Tsao SW Chan AT The activity of mTOR inhibitor RAD001 (everolimus) in nasopharyngeal carcinoma and cisplatin-resistant cell lines Invest New Drugs 2010 28 4 413 420 10.1007/s10637-009-9269-x 19471857

[B39] Liu Y Chen LH Yuan YW Li QS Sun AM Guan J Activation of AKT is associated with metastasis of nasopharyngeal carcinoma Tumour Biol 2012 33 1 241 245 10.1007/s13277-011-0272-4 22116667

[B40] Yip WK Seow HF Activation of phosphatidylinositol 3-kinase/Akt signaling by EGF downregulates membranous E-cadherin and beta-catenin and enhances invasion in nasopharyngeal carcinoma cells Cancer Lett 2012 318 2 162 172 10.1016/j.canlet.2011.12.018 22182447

[B41] Krishna SM Kattoor J Balaram P Down regulation of adhesion protein E-cadherin in epstein-barr virus infected nasopharyngeal carcinomas Cancer Biomark 2005 1 6 271 277 17192051 10.3233/cbm-2005-1602

[B42] Galera-Ruiz H Rios MJ Gonzalez-Campora R de Miguel M Carmona MI Moreno AM Galera-Davidson H The cadherin-catenin complex in nasopharyngeal carcinoma Eur Arch Otorhinolaryngol 2011 268 9 1335 1341 10.1007/s00405-010-1464-z 21240516 PMC3149677

[B43] Zheng Z Pan J Chu B Wong YC Cheung AL Tsao SW Downregulation and abnormal expression of E-cadherin and beta-catenin in nasopharyngeal carcinoma: close association with advanced disease stage and lymph node metastasis Hum Pathol 1999 30 4 458 466 10.1016/S0046-8177(99)90123-5 10208469

[B44] Dabholkar M Bostick-Bruton F Weber C Bohr VA Egwuagu C Reed E ERCC1 and ERCC2 expression in malignant tissues from ovarian cancer patients J Natl Cancer Inst 1992 84 19 1512 1517 10.1093/jnci/84.19.1512 1433335

[B45] Lee HW Hwang YH Han JH Choi JH Kang SY Jeong SH Ann MS Oh YT Kim JH Kim CH High expression of excision repair cross-complementation group 1 protein predicts poor outcome in patients with nasopharyngeal cancer Oral Oncol 2010 46 3 209 213 10.1016/j.oraloncology.2009.12.007 20153243

[B46] Song LB Li J Liao WT Feng Y Yu CP Hu LJ Kong QL Xu LH Zhang X Liu WL The polycomb group protein Bmi-1 represses the tumor suppressor PTEN and induces epithelial-mesenchymal transition in human nasopharyngeal epithelial cells J Clin Invest 2009 119 12 3626 3636 10.1172/JCI39374 19884659 PMC2786794

[B47] Xu X Yang H Huo X [Expression and significance of PTEN in nasopharyngeal carcinoma] Lin Chuang Er Bi Yan Hou Ke Za Zhi 2004 18 11 658 659 15715408

[B48] Peng JP Chang HC Hwang CF Hung WC Overexpression of cyclooxygenase-2 in nasopharyngeal carcinoma and association with lymph node metastasis Oral Oncol 2005 41 9 903 908 10.1016/j.oraloncology.2005.05.003 16054423

[B49] Liu Y Zhao S Liu G Li Z Sun Z Jiang W [The value of cyclooxygenase-2 to predict the effect of radiotherapy in nasopharyngeal carcinoma] Lin Chung Er Bi Yan Hou Tou Jing Wai Ke Za Zhi 2007 21 5 199 202 17536451

[B50] Tan KB Putti TC Cyclooxygenase 2 expression in nasopharyngeal carcinoma: immunohistochemical findings and potential implications J Clin Pathol 2005 58 5 535 538 10.1136/jcp.2004.021923 15858127 PMC1770665

[B51] Kim YJ Go H Wu HG Jeon YK Park SW Lee SH Immunohistochemical study identifying prognostic biomolecular markers in nasopharyngeal carcinoma treated by radiotherapy Head Neck 2011 33 10 1458 1466 10.1002/hed.21611 21928418

[B52] Loong SL Hwang JS Li HH Wee JT Yap SP Chua ML Fong KW Tan TW Weak expression of cyclooxygenase-2 is associated with poorer outcome in endemic nasopharyngeal carcinoma: analysis of data from randomized trial between radiation alone versus concurrent chemo-radiation (SQNP-01) Radiat Oncol 2009 4 23 10.1186/1748-717X-4-23 19591688 PMC2715417

[B53] Pan J Kong L Lin S Chen G Chen Q Lu JJ The clinical significance of coexpression of cyclooxygenases-2, vascular endothelial growth factors, and epidermal growth factor receptor in nasopharyngeal carcinoma Laryngoscope 2008 118 11 1970 1975 10.1097/MLG.0b013e3181805134 18758376

[B54] Soo R Putti T Tao Q Goh BC Lee KH Kwok-Seng L Tan L Hsieh WS Overexpression of cyclooxygenase-2 in nasopharyngeal carcinoma and association with epidermal growth factor receptor expression Arch Otolaryngol Head Neck Surg 2005 131 2 147 152 10.1001/archotol.131.2.147 15723947

[B55] Fendri A Khabir A Hadhri-Guiga B Sellami-Boudawara T Ghorbel A Daoud J Frikha M Jlidi R Gargouri A Mokdad-Gargouri R Overexpression of COX-2 and LMP1 are correlated with lymph node in Tunisian NPC patients Oral Oncol 2008 44 7 710 715 10.1016/j.oraloncology.2007.09.006 18061524

[B56] Murono S Inoue H Tanabe T Joab I Yoshizaki T Furukawa M Pagano JS Induction of cyclooxygenase-2 by epstein-barr virus latent membrane protein 1 is involved in vascular endothelial growth factor production in nasopharyngeal carcinoma cells Proc Natl Acad Sci USA 2001 98 12 6905 6910 10.1073/pnas.121016998 11381123 PMC34451

[B57] Wan XB Long ZJ Yan M Xu J Xia LP Liu L Zhao Y Huang XF Wang XR Zhu XF Inhibition of Aurora-A suppresses epithelial-mesenchymal transition and invasion by downregulating MAPK in nasopharyngeal carcinoma cells Carcinogenesis 2008 29 10 1930 1937 10.1093/carcin/bgn176 18667445

[B58] Sheu LF Chen A Tseng HH Leu FJ Lin JK Ho KC Meng CL Assessment of p53 expression in nasopharyngeal carcinoma Hum Pathol 1995 26 4 380 386 10.1016/0046-8177(95)90137-X 7705815

[B59] Chow LW Khoo US Yuen AP Wei WI Expression of p53 in recurrent nodal metastasis from nasopharyngeal carcinoma (NPC) Eur J Surg Oncol 1997 23 5 415 418 10.1016/S0748-7983(97)93721-5 9393569

[B60] Liu QY Han AJ You SY Dong Y Yang QX Wu JH Li MF [Correlation of epstein-barr virus-encoded latent membrane protein 1 (LMP1) to fascin and phosphorylated Stat3 in nasopharyngeal carcinoma] Ai Zheng 2008 27 10 1070 1076 18851787

[B61] Cao XJ Hao JF Yang XH Xie P Liu LP Yao CP Xu J Prognostic value of expression of EGFR and nm23 for locoregionally advanced nasopharyngeal carcinoma Med Oncol 2012 29 1 263 271 10.1007/s12032-010-9782-y 21221850

[B62] Taheri-Kadkhoda Z Magnusson B Svensson M Mercke C Bjork-Eriksson T Expression modes and clinical manifestations of latent membrane protein 1, Ki-67, cyclin-B1, and epidermal growth factor receptor in nonendemic nasopharyngeal carcinoma Head Neck 2009 31 4 482 492 10.1002/hed.21002 19132724

[B63] Kung CP Meckes DG Jr Raab-Traub N Epstein-Barr virus LMP1 activates EGFR, STAT3, and ERK through effects on PKCdelta J Virol 2011 85 9 4399 4408 10.1128/JVI.01703-10 21307189 PMC3126279

[B64] Ruan L Li XH Wan XX Yi H Li C Li MY Zhang PF Zeng GQ Qu JQ He QY Analysis of EGFR signaling pathway in nasopharyngeal carcinoma cells by quantitative phosphoproteomics Proteome Sci 2011 9 35 10.1186/1477-5956-9-35 21711528 PMC3141626

[B65] Ma BB Kam MK Leung SF Hui EP King AD Chan SL Mo F Loong H Yu BK Ahuja A A phase II study of concurrent cetuximab-cisplatin and intensity-modulated radiotherapy in locoregionally advanced nasopharyngeal carcinoma Ann Oncol 2012 23 5 1287 1292 10.1093/annonc/mdr401 21948811

[B66] Vermorken JB Mesia R Rivera F Remenar E Kawecki A Rottey S Erfan J Zabolotnyy D Kienzer HR Cupissol D Platinum-based chemotherapy plus cetuximab in head and neck cancer N Engl J Med 2008 359 11 1116 1127 10.1056/NEJMoa0802656 18784101

[B67] Hui EP Chan AT Pezzella F Turley H To KF Poon TC Zee B Mo F Teo PM Huang DP Coexpression of hypoxia-inducible factors 1alpha and 2alpha, carbonic anhydrase IX, and vascular endothelial growth factor in nasopharyngeal carcinoma and relationship to survival Clin Cancer Res 2002 8 8 2595 2604 12171890

[B68] Wakisaka N Wen QH Yoshizaki T Nishimura T Furukawa M Kawahara E Nakanishi I Association of vascular endothelial growth factor expression with angiogenesis and lymph node metastasis in nasopharyngeal carcinoma Laryngoscope 1999 109 5 810 814 10.1097/00005537-199905000-00024 10334236

[B69] Shi XY Hu GQ Yuan XL Li HY Liu YQ Ma D [Relationship between VEGF-C expression and nasopharyngeal carcinoma proliferation and metastasis] Zhonghua Zhong Liu Za Zhi 2006 28 5 364 367 17045002

[B70] Yi X Tang A Qin Y Wen W Zhao W [Expression and relationship of EBV LMP1, COX-2 and VEGF-C in nasopharyngeal carcinoma] Lin Chung Er Bi Yan Hou Tou Jing Wai Ke Za Zhi 2010 24 3 126 128 20429385

[B71] Lee NY Zhang Q Pfister DG Kim J Garden AS Mechalakos J Hu K Le QT Colevas AD Glisson BS Addition of bevacizumab to standard chemoradiation for locoregionally advanced nasopharyngeal carcinoma (RTOG 0615): a phase 2 multi-institutional trial Lancet Oncol 2012 13 2 172 180 10.1016/S1470-2045(11)70303-5 22178121 PMC4985181

[B72] Sabatini DM mTOR and cancer: insights into a complex relationship Nat Rev Cancer 2006 6 9 729 734 10.1038/nrc1974 16915295

[B73] Chen J Hu CF Hou JH Shao Q Yan LX Zhu XF Zeng YX Shao JY Epstein-Barr virus encoded latent membrane protein 1 regulates mTOR signaling pathway genes which predict poor prognosis of nasopharyngeal carcinoma J Transl Med 2010 8 30 10.1186/1479-5876-8-30 20338061 PMC2861642

[B74] Curtis C Shah SP Chin SF Turashvili G Rueda OM Dunning MJ Speed D Lynch AG Samarajiwa S Yuan Y The genomic and transcriptomic architecture of 2,000 breast tumours reveals novel subgroups Nature 2012 486 7403 346 352 22522925 10.1038/nature10983 PMC3440846

[B75] Huang C Tang H Zhang W She X Liao Q Li X Wu M Li G Integrated analysis of multiple gene expression profiling datasets revealed novel gene signatures and molecular markers in nasopharyngeal carcinoma Cancer Epidemiol Biomarkers Prev 2012 21 1 166 175 10.1158/1055-9965.EPI-11-0593 22068284

[B76] Wang HY Sun BY Zhu ZH Chang ET To KF Hwang JS Jiang H Kam MK Chen G Cheah SL Eight-signature classifier for prediction of nasopharyngeal [corrected] carcinoma survival J Clin Oncol 2011 29 34 4516 4525 10.1200/JCO.2010.33.7741 22025164

[B77] Espina V Edmiston KH Heiby M Pierobon M Sciro M Merritt B Banks S Deng J VanMeter AJ Geho DH A portrait of tissue phosphoprotein stability in the clinical tissue procurement process Mol Cell Proteomics 2008 7 10 1998 2018 10.1074/mcp.M700596-MCP200 18667411 PMC2559936

