# Supplementary figures and images for: Expression profiling of 21 biomolecules in locally advanced nasopharyngeal carcinomas of Caucasian patients
Source: BMC Clin Pathol. 2013 Jan 29;13:1. doi: 10.1186/1472-6890-13-1 (PMC3563444; doi:10.1186/1472-6890-13-1)

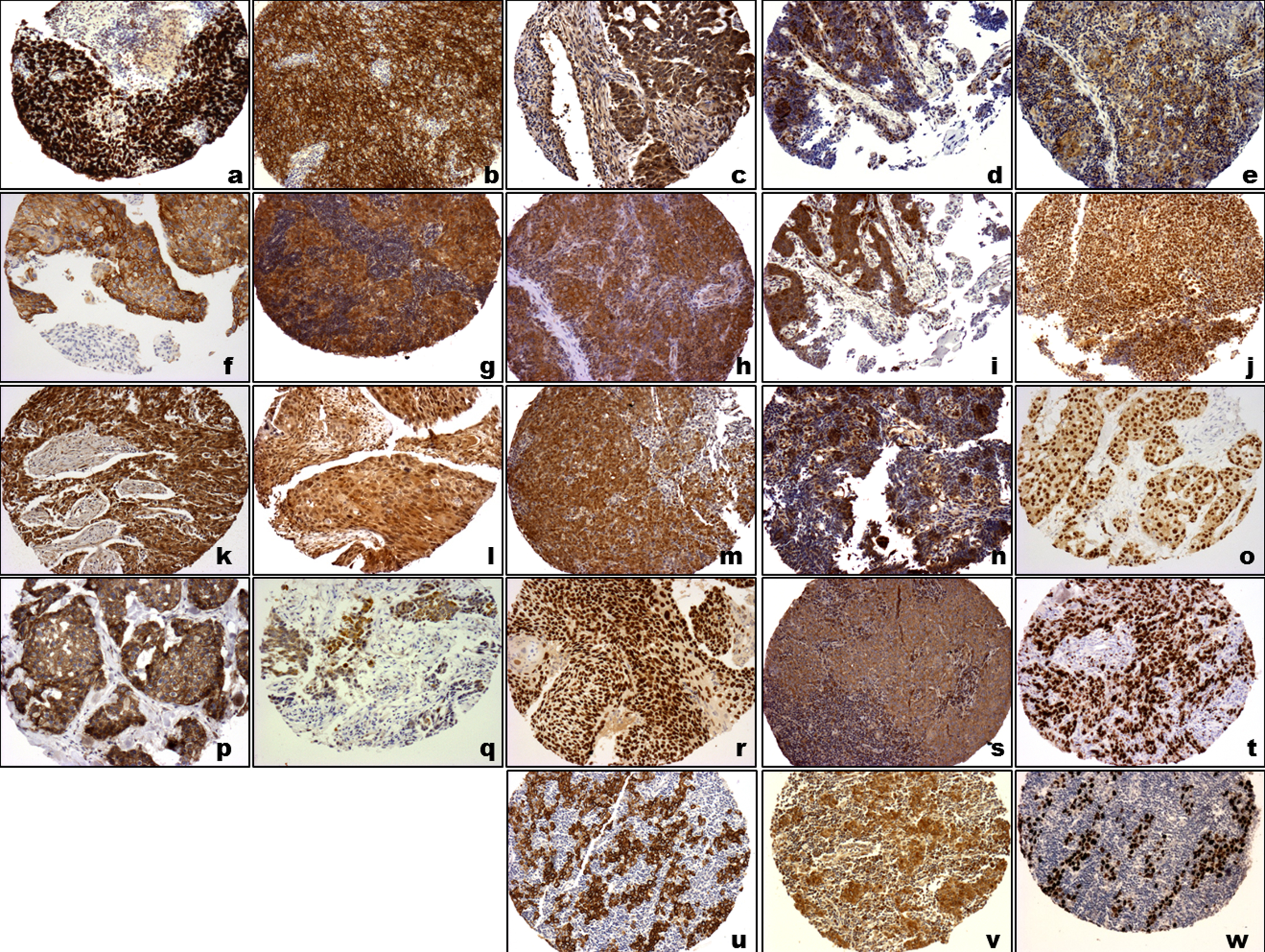

Supplement: Additional file 1 — Figure S1. Protein expression detected by IHC and CISH in tissue microarrays from nasopharyngeal carcinoma cases. (a) p53; (b) EGFR; (c) COX-2; (d) VEGF-A; (e) MAPT; (f) E-cadherin; (g) PTEN; (h) p-GSK-3β; (i) VEGF-C; (j) ERCC1; (k) Fascin-1; (l) p-AKT; (m) p-p44/42 MAPK; (n) VEGFR-2; (o) Cyclin D1; (p) P-cadherin; (q) p-mTOR; (r) p63; (s) VEGFR-3; (t) Ki67; (u) Multi-Cytokeratin; (v) mRNA probe (CISH); (w) EBER probe (CISH). Original magnification ×100. [file 1472-6890-13-1-S1.jpeg]

# EBER

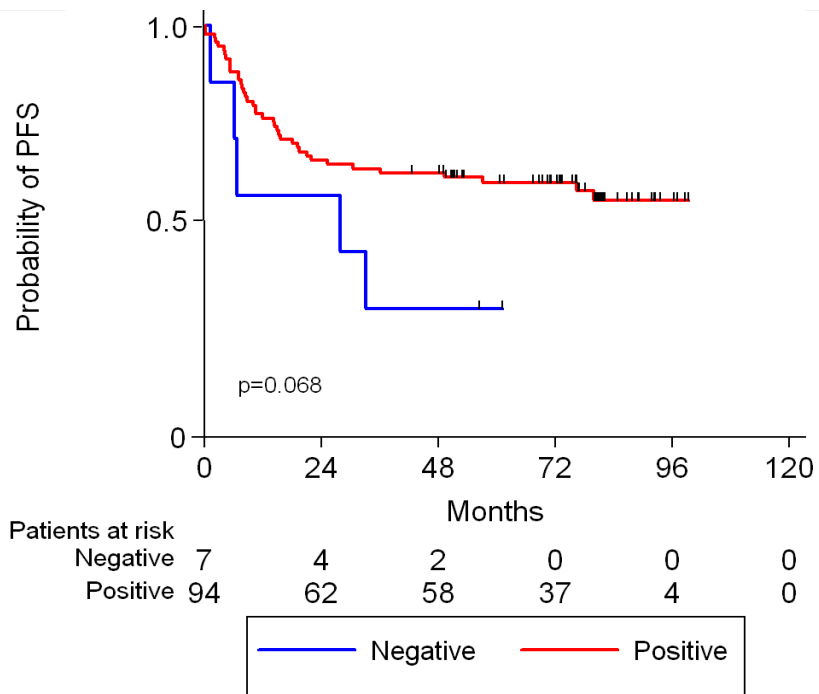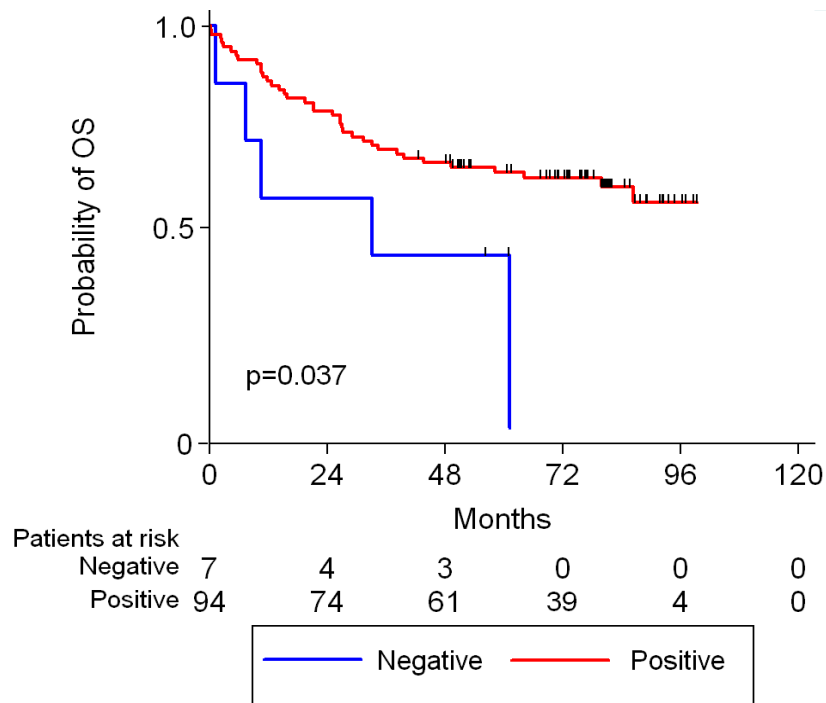

p63

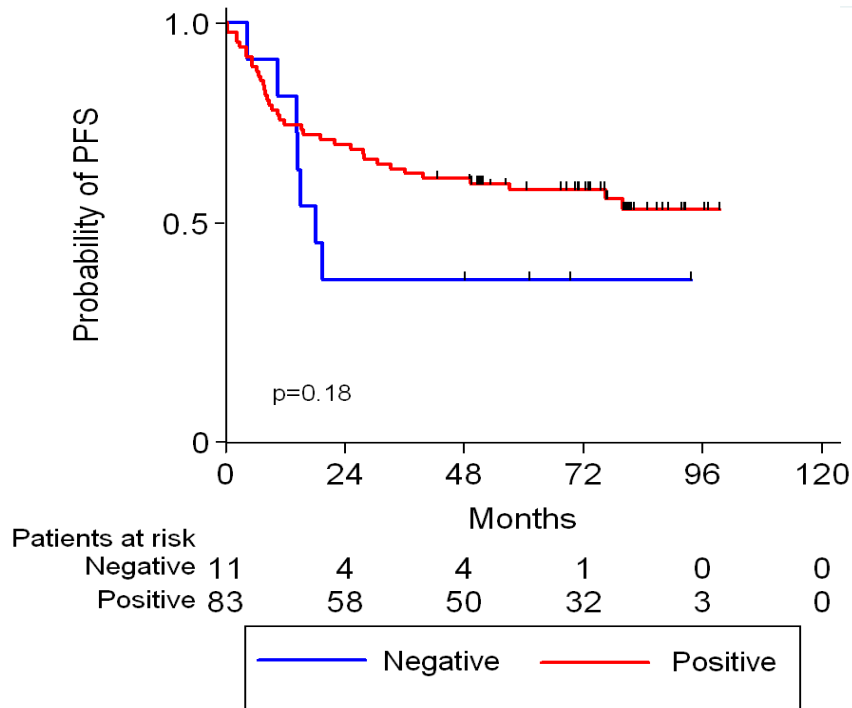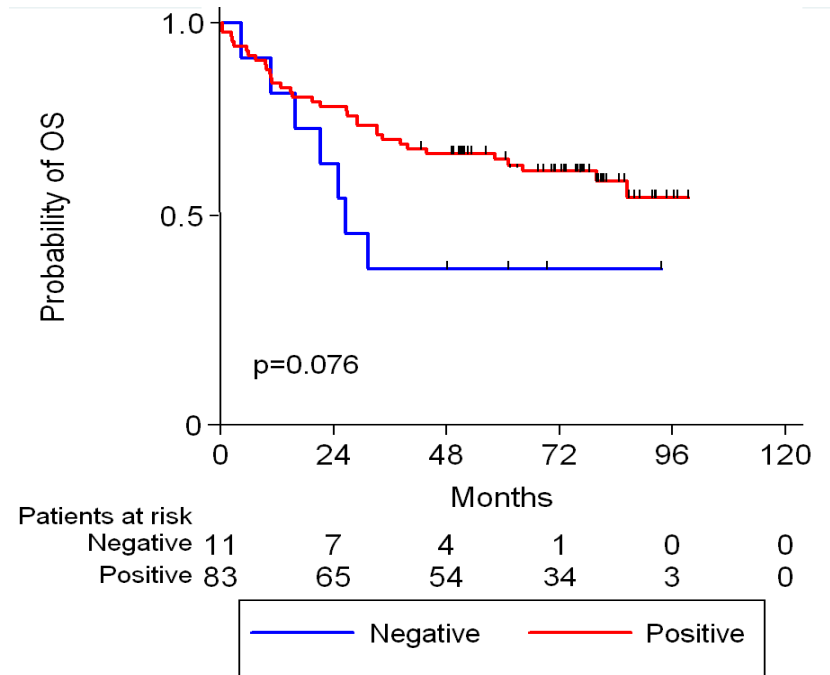

# mTOR

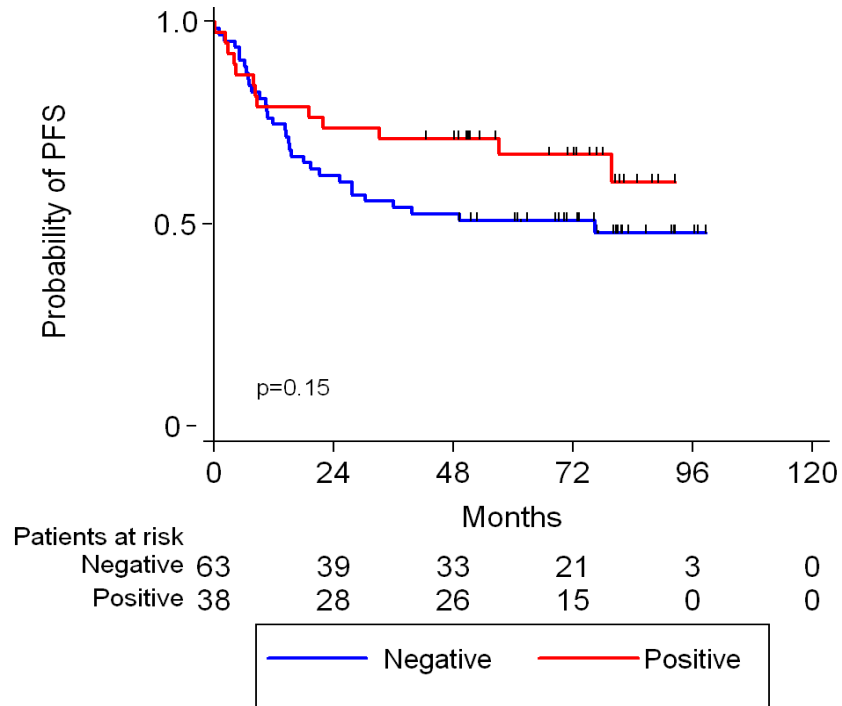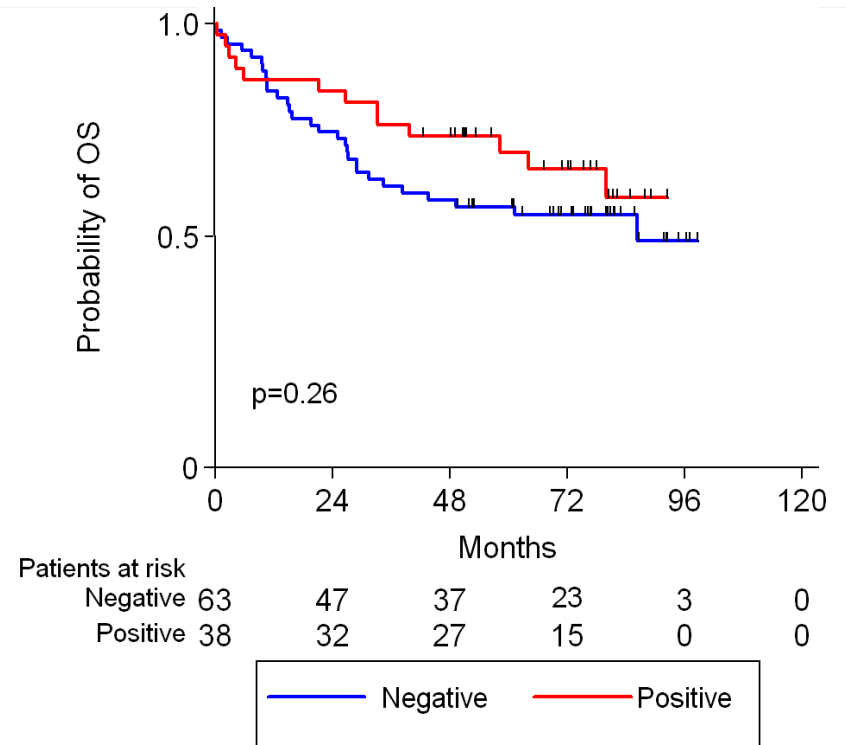

# ERCC1

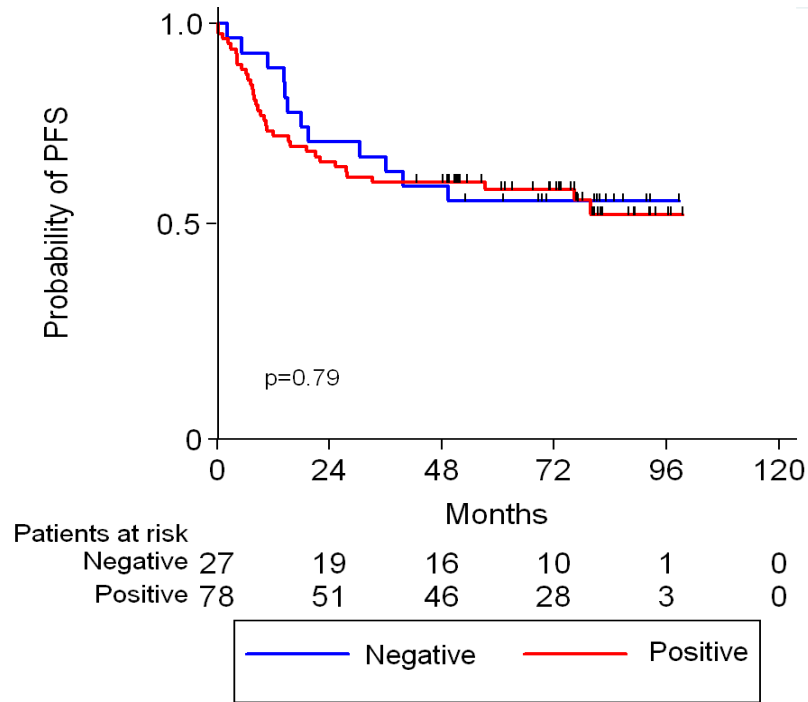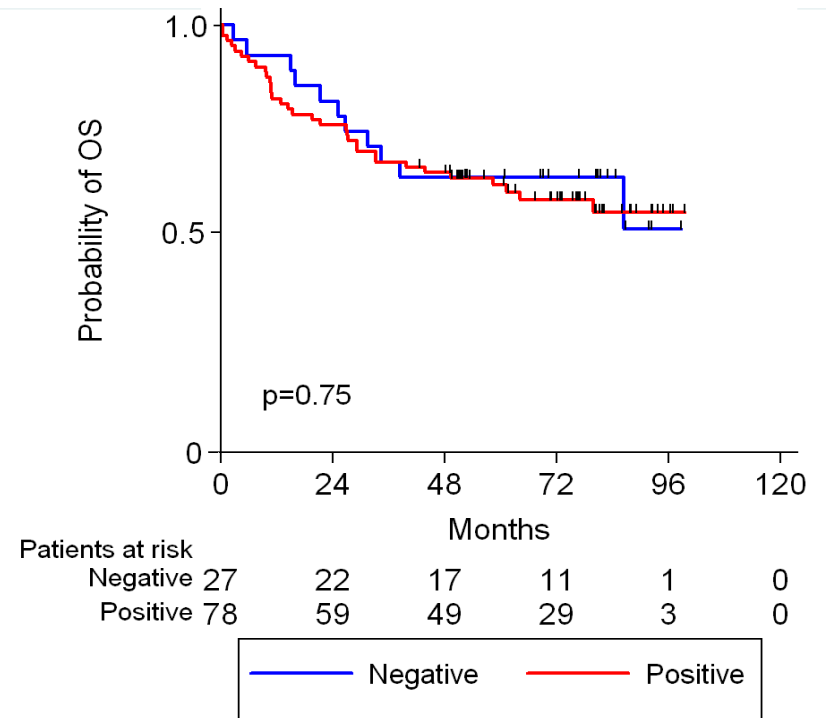

# Cyclin D1

all patients, irrespective of treatment Groups

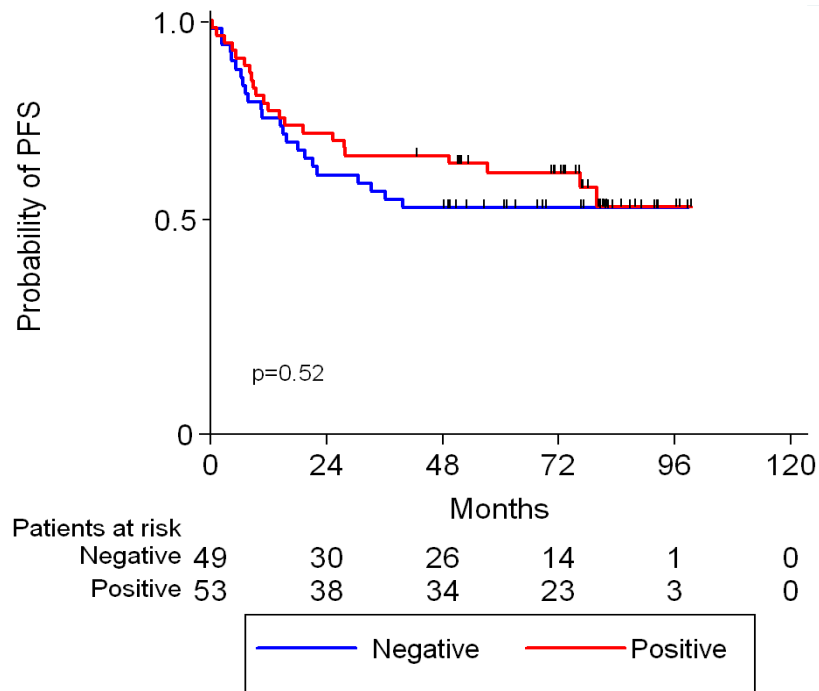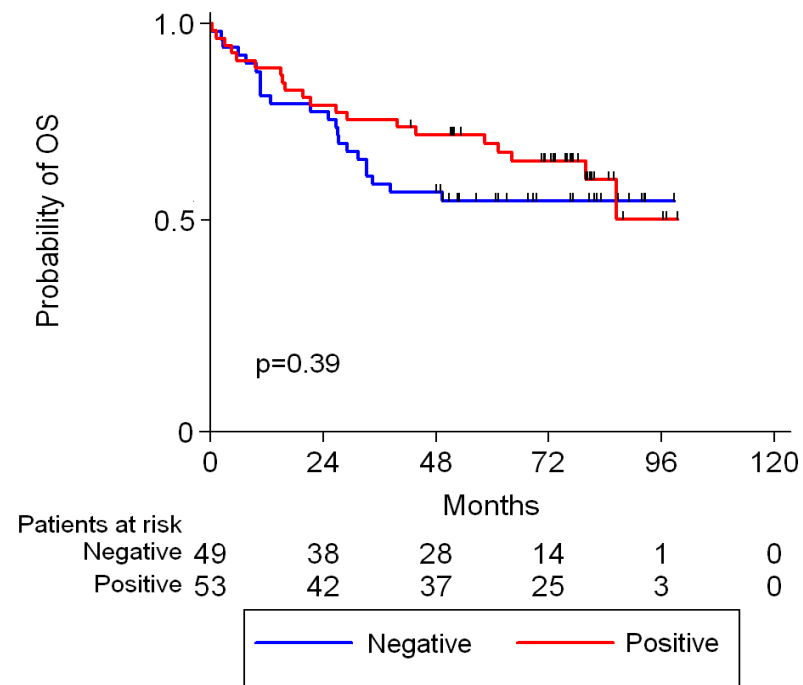

Supplement: Additional file 4 — Figure S2. Prognostic significance of EBER, p63, mTOR, ERCC1 and Cyclin D1 protein expression for progression-free and overall survival (Log-Rank test). For Cyclin D1, all patients have been included, irrespective of the treatment administered. [file 1472-6890-13-1-S4.pdf]
